# MEIS1 inhibits clear cell renal cell carcinoma cells proliferation and in vitro invasion or migration

**DOI:** 10.1186/s12885-017-3155-2

**Published:** 2017-03-07

**Authors:** Jie Zhu, Liang Cui, Axiang Xu, Xiaotao Yin, Fanglong Li, Jiangping Gao

**Affiliations:** 1 Department of Urology, Chinese PLA Medical School/Chinese PLA General Hospital, Beijing, 100853 People’s Republic of China; 2 Department of Urology, Civil Aviation General Hospital/Civil Aviation Medical College of Peking University, Beijing, 100123 People’s Republic of China

**Keywords:** MEIS1, Clear cell renal cell carcinoma, Proliferation, in vitro invasion or migration

## Abstract

**Background:**

Myeloid ecotropic viral integration site 1 (MEIS1) protein plays a synergistic causative role in acute myeloid leukemia (AML). However, MEIS1 has also shown to be a potential tumor suppressor in some other cancers, such as non-small-cell lung cancer (NSCLC) and prostate cancer. Although multiple roles of MEIS1 in cancer development and progression have been identified, there is an urgent demand to discover more functions of this molecule for further therapeutic design.

**Methods:**

MEIS1 was overexpressed via adenovirus vector in clear cell renal cell carcinoma (ccRCC) cells. Western blot and real-time qPCR (quantitative Polymerase Chain Reaction) was performed to examine the protein and mRNA levels of MEIS1. Cell proliferation, survival, in vitro migration and invasion were tested by MTT, colony formation, soft-agar, transwell (in vitro invasion/migration) assays, and tumor in vivo growthwas measured on nude mice model. In addition, flow-cytometry analysis was used to detect cell cycle arrest or non-apoptotic cell death of ccRCC cells induced by MEIS1.

**Results:**

MEIS1 exhibits a decreased expression in ccRCC cell lines than that in non-tumor cell lines. MEIS1 overexpression inhibits ccRCC cells proliferation and induces G1/S arrest concomitant with marked reduction of G1/S transition regulators, Cyclin D1 and Cyclin A. Moreover, MEIS1-1 overexpression also induces non-apoptotic cell death of ccRCC cells via decreasing the levels of pro-survival regulators Survivin and BCL-2. Transwell migration assay (TMA) shows that MEIS1 attenuates in vitro invasion and migration of ccRCC cells with down-regulated epithelial-mesenchymal transition (EMT) process. Further, in nude mice model, MEIS1 inhibits the in vivo growth of Caki-1 cells.

**Conclusions:**

By investigating the role of MEIS1 in ccRCC cells’ survival, proliferation, anchorage-independent growth, cell cycle progress, apoptosis and metastasis, in the present work, we propose that MEIS1 may play an important role in clear cell renal cell carcinoma (ccRCC) development.

## Background

As a member of TALE (Triple amino acid loop extension) transcription factors, MEIS1 contains a homeodomain which is generally recognized be important for its roles in cell growth and differentiation during vertebrate embryogenesis [1–3]. MEIS1 also plays a critical role in development and stem cells regulation [4–6]. Various genetic alterations occur in AML that lead to overexpression of the MEIS/HOXA9 complex, which plays a synergistic causative role in acute myeloid leukemia (AML) development [7–10]. However, a range of recent work also revealed that MEIS1 would also be found as a negative regulator in some other cancers, e.g. non-small-cell lung cancer or prostate cancer [11–13]. In these cancers, MEIS1 would function via inhibiting cell proliferation and inducing cell cycle arrest [11–13]. High level of MEIS1 was detected in normal prostate compared with prostate tumor tissues; indicating that it would be a useful biomarker or even a therapeutic target of human prostate carcinoma [13]. Rad et al reported that MEIS1 level is inversely correlated with tumor metastasis and tumor staging of esophageal squamous cell carcinoma [14]. Xu et al showed that MEIS1 would interact with Hox (homeobox protein family) and Pbx1 (pre-B-cell leukemia homeobox 1) [15]. Our previous work also indicated that MEIS1 is a negative regulator of androgen receptor (AR) pathway [16]. To unmask thorough roles by which MEIS1 affect cancer physiology, more studies should be done.

The ccRCC is one of the most common malignancies, and it is the second leading cause of death among patients suffering from urologic tumors [17, 18]. Nowadays, partial nephrectomy is the most effective treatment for local ccRCC [18]. As to chemotherapy and radiotherapy, the prognosis of advanced metastatic ccRCCs still remains very poor because tumor tissue has low sensitivity to those therapeutic agents [17–20]. Although certain kinase inhibitors have been used in clinical application, such as sorafenib and sunitinib, several problems have been raised during recent trials, i.e. the risk of adverse events and un-anticipated efficacy-loss, which may due to the off target effects of current agents [19, 20]. Therefore, it is of great importance to identify novel molecular targets for ccRCC treatment.

In this study, we find that MEIS1 is less expressed in some ccRCC cell lines and ccRCC specimens than in non-tumor kidney cells and specimens. Overexpression of MEIS1 significantly inhibits cell survival and induces cell cycle G1/S phase arrest of ccRCC cells 786-O and Caki-1. Furthermore, we demonstrate that reestablishment of MEIS1 expression in high aggressive ccRCC cell line Caki-1 leads to dramatic in vitro cell invasion and migration reduction and decreased EMT process. Moreover, overexpression of MEIS1 also inhibits in vivo growth of Caki-1 cells in mouse model.

## Methods

### Plasmids and adenovirus vector preparation

The MEIS1 expression vector or its small interfering RNA (siRNA) was as previously described [16]. Adenovirus vectors of MEIS1 were generated following the methods as described by Niu et al. [21]. Briefly, full length cDNA of *Meis1* gene was cloned into pShuttle-CMV vector. Then, pAdEasy-1 vector and pShuttle-*Meis1* vector was co-transformed into BJ5183 cells to produce the recombinant adenovirus vector pAd-control or pAd-MEIS1. For packaging step, pAd-control or pAd-MEIS1 was transfected into AD-293 cells and then purified with a cesium chloride gradient. All vectors were confirmed by Sanger sequencing.

### Cell culture and reagents

Human ccRCC cell lines 786-O or Caki-1 (a high metastatic cell line), and non-tumor cell lines HEK293 (a human embryonic kidney cell line) or HKC (a human kidney non-tumor cell line) were as previously described [18]. 786-O, Caki-1 and HKC cells were cultured in complete DMEM (Invitrogen, Carlsbad, CA, USA), and HEK293 was cultured in RPMI-1640 medium (Invitrogen, Carlsbad, CA, USA) in a sterile incubator maintained at 37 °C with 5% CO_2_.

### Cell growth and colony formation assays

For measuring proliferation, Caki-1 or 786-O cells, which were infected with Ad-control or Ad-MEIS1, were seeded in 96-well plates (Corning, NY, USA), incubated for 1, 2, 3 and 4 days, and the cells were analyzed for MTT assays [22]. HKC cells were transfected with siRNA of MEIS1 and then harvested for MTT analysis.

For colony formation, infected ccRCC cells were seeded in 6-well plates at 500 cells per well [23]. Two to four weeks later, colonies were fixed with 4% paraformaldehyde and stained with 0.5% (W/W) crystal violet (diluted in phosphate buffer saline, PBS) for 30 min. Next, cells were harvested and measured by a multifunctional micro-plate reader at 546 nm. The relative colony number (relative survival cell number) = *O.D. 546* administration group / *O.D. 546* control group. HKC cells, which were transfected with siRNA of MEIS1, were also measured by colony formation assays.

### Cell cycle analysis

Cell cycle was carried out by flow-cytometry following the instructions as previously described by Chen et al [24]. ccRCC cells, which were infected with Ad-control or Ad-MEIS1, were fixed in 70% ethanol for 18-24 h. Next, cells were washed with pre-cold PBS for three times and incubated with RNase A (0.2 mg/mL) diluted in pre-cold PBS. Then, PI (propidium Iodide) was added. Samples were analyzed by FACScalibur Flow Cytometer (Becton Dickinson, Bioscience, USA).

### Cell death analysis

Caki-1 or 786-O cells, which were infected with Ad-control or Ad-MEIS1, were harvested and labelled with PI and FITC-Annexin V according to the manufacturer’s instructions (Becton Dickinson, Biosciences, USA). A minimum of 2000 events for each sample were collected and analyzed using a FACScalibur Flow Cytometer (Becton Dickinson, Biosciences, USA).

### Real-time PCR (qPCR)

Total RNA samples of cells or clinical specimens were obtained by previously [18, 25]. The qPCR was performed according to the manufacturer’s instructions (Applied Biosystems, Foster City, CA, USA) using total RNA sample as templates. The primers used to amplify MEIS1 expression are: forward: 5’-TCCCAA AGTAGCCACCAATATC-3’; and reverse: 5’-CTGTATCTGTGCCAAC TGCTT-3’

### Anchorage-independent growth

Caki-1 or 786-O cells, which were infected with Ad-control or Ad-MEIS1, were plated into 6-well plates (1000 cells per well) (Corning, Corning, NY, USA), with a bottom layer of 1.0% low melting temperature agar in DMEM and a top layer of 0.3% agar in DMEM. Colony number was manifested as the mean ± SD of three independent experiments scored after 3-4 weeks of growth [26].

### Transwell migration assays (in vitro invasion or migration)

Caki-1 cells, which were infected with Ad-control or Ad-MEIS1, were analyzed by transwell migration assays performed in 24-well plates chamber (Corning, NY, USA) by fitted with a polyethylene terephthalate filter membrane with 8 μm pores. For invasion, the top chambers was coated with 30 μl ECM (Extracellular matrix) gel (Sigma, USA) diluted with serum free RPMI-1640 in 1:5 dilution for more than 4 h at 37 °C. Then, chambers were filled with 0.2 ml of cells (5 × 10^5^ cells per ml) in serum-free medium, and the bottom chambers were filled with 0.25 ml of medium with 10% FBS. The cells were incubated in the trans-wells at 37 °C in 5% CO_2_ for 12 h. For migration, the cells were incubated in chambers without ECM coated. After 10-15 h (for invasion) or 4-6 h (for migration), chamber membrane was fixed with 4% paraformaldehyde and stained with crystal violet. The relative invading cells were measured according to the instructions provided [27, 28]. Values were presented as the mean ± SD of triplicated experiments.

### Antibodies and western blot

Antibodies against MEIS1, Cyclin D1, Cycline A, cIAP1/2, Survivin, E-cadherin, N-cadherin, Vimentin, BAX and GAPDH were obtained from Santa Cruz Biotechnology (Santa Cruz Biotech, CA, USA). Polyclonal IgG conjugated with horseradish peroxidase (HRP) was obtained from Sigma (St. Louis, MO, USA). Total protein samples were performed by SDS-PAGE and trans-printed to poly-vinylidene fluoride (PVDF) membranes (Millipore, Billerica, MA). Then, membranes were blocked with 5% BSA in TBST buffer and then incubated 2 h at 37 °C with primary antibody against MEIS1 (1:1000), Cyclin D1 (1:500), Cyclin A (1:500), cIAP-1 (1:1000), cIAP-2 (1:1000), Survivin (1:2000), E-cadherin (1:1000), N-cadherin (1:1000), Vimentin (1:2000), BAX (1:500) and GAPDH (1:5000) diluted in TBST containing 5% BSA and subsequently washed three times in TBST for 5 min each. Then membranes were incubated with the HRP-conjugated secondary antibodies (1:5000) after being washed three times in TBST for 5 min each. At last, blots were developed with enhanced chemiluminescence reagents by X-ray films. The blots were performed on three experiments with similar results.

### In vivo analysis

In vivo nude mice model was created via xenotransplantation of Caki-1 cells into 4-5 week-old nude mice (6 animals per group) [29]. Caki-1 cells, which were infected with Ad-control or AD-MEIS1, were injected with 5 × 10^5^ cells re-suspended in PBS or normal/physiological saline. Tumor volumes were measured every week by measuring length and width. Volumes of tumor were calculated: (width^2^ × length) / 2 [29]. Animal Experiment Committee of General Hospital of Chinese PLA approved all protocols for treating animals, and the in vivo studies were carried out in accordance with the U.K. Animals (Scientific Procedures) Act, 1986 and associated guidelines.

### Statistical analysis

The WB results were analyzed by the ALPHA INNOTECH analysis software. The relative expression level was calculated: (indicated group protein expression level/loading control expression level)/(control group protein expression level/loading control expression level). All statistical significance analyses were performed using SPSS statistical software. *P*-value of <0.05 was considered statistically significant. Statistical significance in cell growth assays was analyzed by Bonferroni correction with or without two-way ANOVA.

## Results

### MEIS1 is expressed in ccRCC cell lines and tissues

**Fig. 1:**
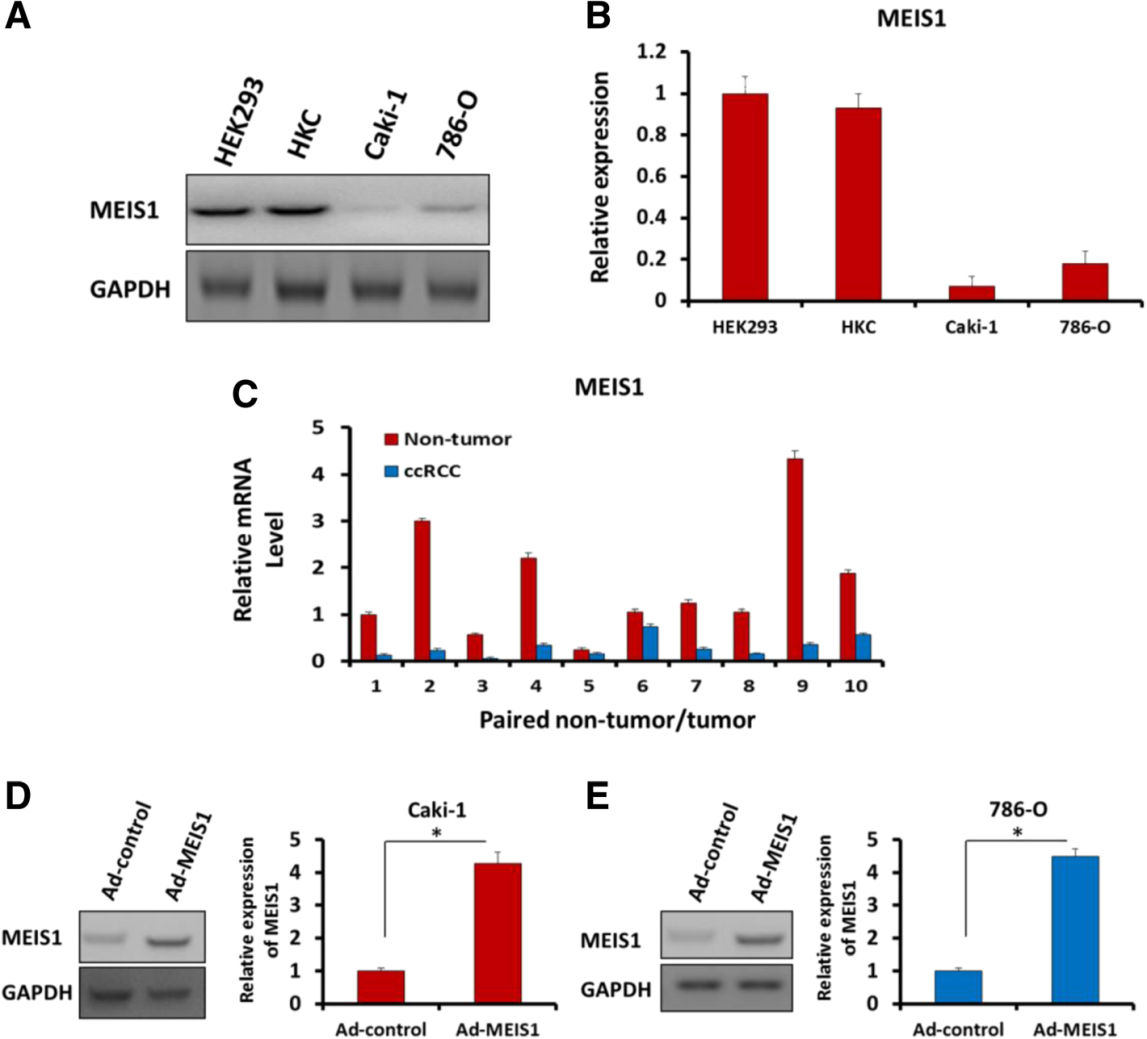
MEIS1 expresses in ccRCC cells 786-O and Caki-1 and kidney non-tumor cells HEK293, and HKC. **a** Total protein extracted from the indicated cell lines were analyzed by WB. GAPDH was chosen as a loading control. **b** The relative protein level was shown as mean ± SD from three independent experiments with similar results. **c** The mRNA level of Meis1 in 10-paired colorectal cancer and matched adjacent was determined by real-time RT-PCR. The ccRCC cells, Caki-1 (**d**) or 786-O (**e**) were infected with Ad-control or Ad-MEIS1. The expression of MEIS1 in ccRCC cells were shown as photographs or relative expression level. *p < 0.05 versus with cells infected Ad-control or Ad-MEIS1

First, the endogenous level of MEIS1 in ccRCC and non-tumor kidney cells was examined by WB. As shown in Fig. 1a, b, high level of MEIS1 was detected in non-tumor cell line, HEK-293 and HKC. In contrast, MEIS1 expression in ccRCC cell lines Caki-1 and 786-O was much lower. Next, the expression of MEIS1 in ccRCC clinical specimens was detected (Fig. 1c). Ten paired ccRCC/non-tumor cDNA samples were analyzed by qPCR. The endogenous level of MEIS1 was significantly lower in ccRCC samples than in adjacent non-neoplastic clinical specimens (Fig. 1c). Our data from cell lines and clinical specimens suggest that loss of MEIS1 may play a role in the progression of ccRCC.

Next, to further study the detailed functions of MEIS1 in ccRCC, we generated MEIS1 expressing adenoviral vectors. Ad-control carries out the empty vector and Ad-MEIS1 contains full length coding sequence of *Meis1* gene. Caki-1 or 786-O cells were infected with Ad-control or Ad-MEIS1. The protein level of MEIS1 was significantly increased upon Ad-MEIS1 infection (Fig. 1d, e).

### MEIS1 suppresses ccRCC cells proliferation

**Fig. 2:**
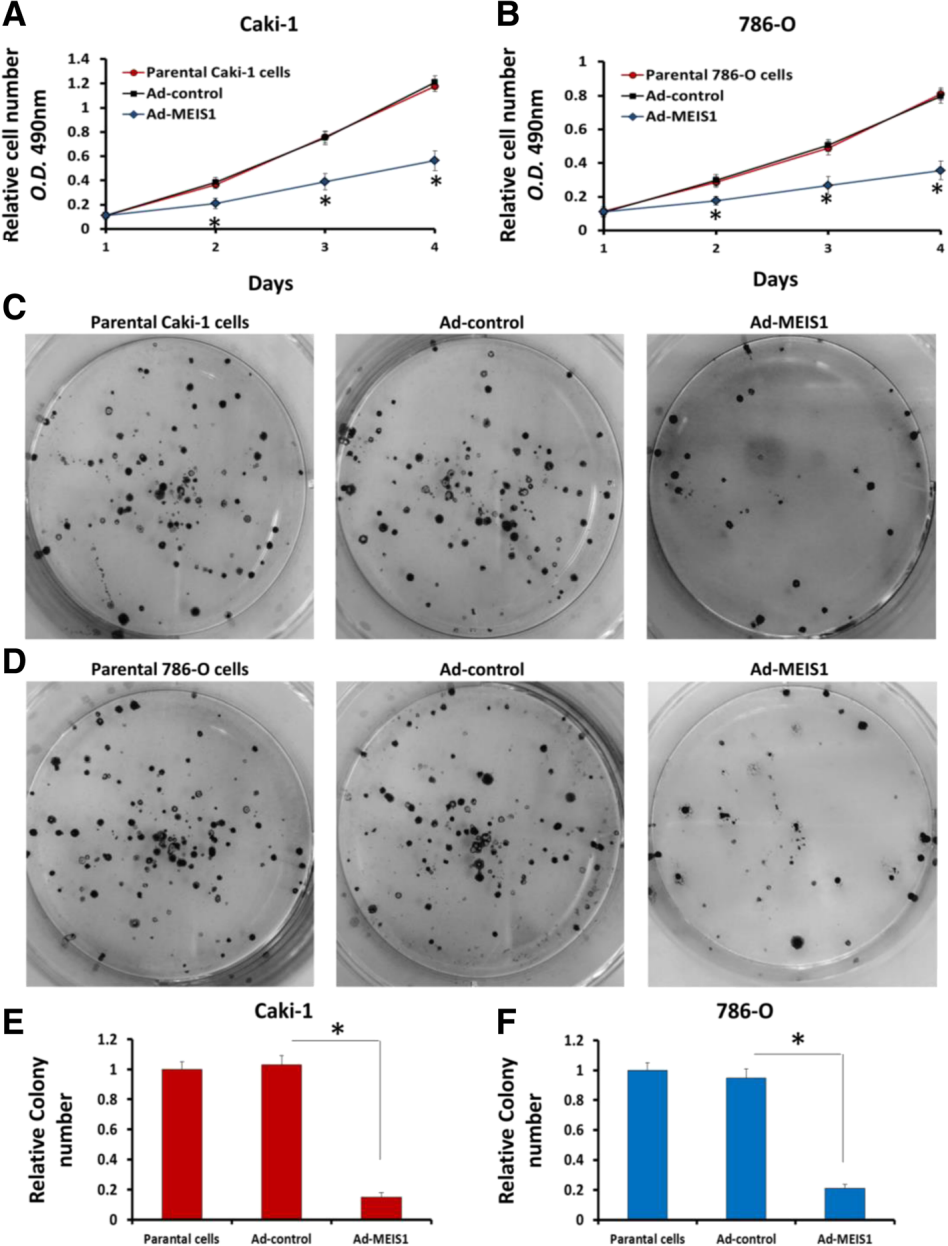
MEIS1 attenuates ccRCC cells proliferation or survival. Caki-1 (**a**) or 786-O (**b**) cells infected with Ad-control, Ad-MEIS1 or the parental cells were grown in regular medium and harvested at indicated time points. Cell number was determined by MTT. Then, colony formation for Caki-1 (**c**) and 786-O (**d**) cells infected with Ad-control or Ad-MEIS1 were measured. All values were shown as photograph (**c**, **d**) or mean ± SD (**e**, **f**) of triplicate measurements with similar results. *p < 0.05 versus with parental cells or cells infected Ad-MEIS1, *p < 0.05 versus with Ad-control or Ad-MEIS1

To test whether MEIS1suppresses ccRCC proliferation, we performed MTT assay. Caki-1 and 786-O cells that were infected with Ad-MEIS1 grew slower than those infected with Ad-controls or their parental cells (Fig. 2a, b). Since there was no significant difference between the parental cells and cells infected with Ad-controls (Fig. 2a, b), our data suggest that MEIS1 overexpression inhibits ccRCC cell growth. Moreover, colony formation assay (CFAs) showed that colony number was reduced in MEIS1 overexpressing Caki-1 or 786-O cells than in control cells (Fig. 2c-f). Together, these results reveal that MEIS1 may attenuate the proliferation and colony formation of ccRCC cells.

**Fig. 3:**
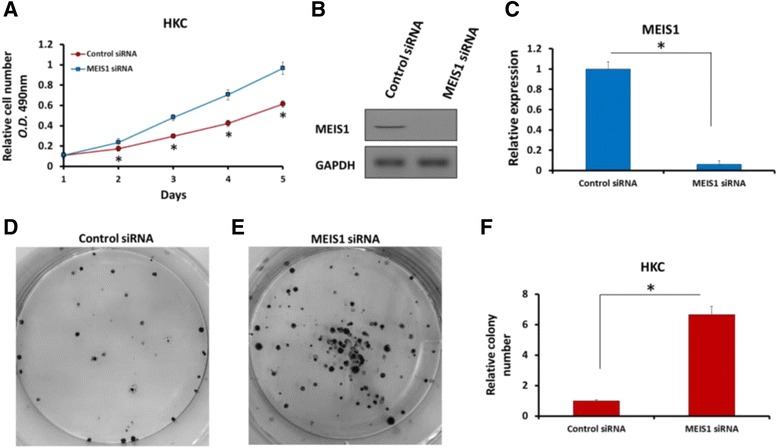
Down-regulation of MEIS1 via siRNA promotes HKC proliferation or survival. HKC cells (**a**) infected with Ad-control or Ad-MEIS1 were grown in regular medium and harvested at the indicated time points. Cell numbers were determined by MTT assay at 490 nm. The expression of MEIS1 was identified by WB (**b**, **c**). Next, colony formation for HKC cells (**d**) was measured. All values were shown as photograph (**d**, **e**) or mean ± SD (**f**) of triplicate measurements with similar results. *p < 0.05 versus with Ad-control or Ad-MEIS1

Next, to confirm MEIS1’s function, we knockdown MEIS1 in HKC cells; HKC is a normal human kidney cell line highly expressing endogenous MEIS1. As shown in Fig. 3, knockdown of MEIS1 via its siRNA enhanced the proliferation (Fig. 3a-c), colony number and size (Fig. 3d-f) of HKC cells. These results confirmed that MEIS1 negatively regulates cell growth and proliferation.

### MEIS1 decreases the anchorage-independent growth of ccRCC cells

**Fig. 4:**
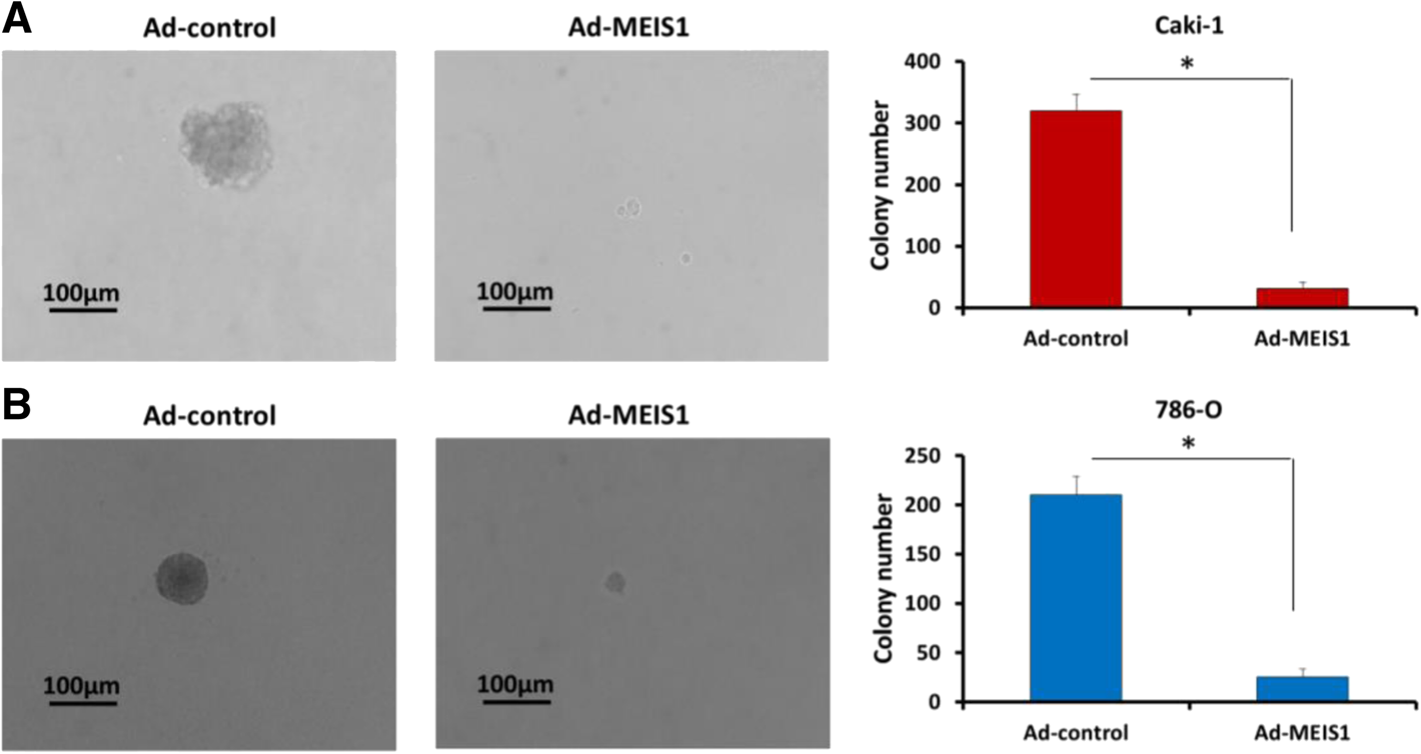
MEIS1 inhibits anchorage-independent growth of ccRCC cells. Caki-1 (**a**) or 786-O (**b**) cells, which were infected with Ad-control or Ad-MEIS1, were analyzed by soft agar. Results were shown as photograph or mean ± SD of triplicate measurements with similar results. *p < 0.05 versus with Ad-control or Ad-MEIS1

To further examine tumor-suppressing activity of MEIS1, soft-agar assay was performed to examine the anchorage-independent growth of ccRCC cells. Our results indicated that MEIS1 overexpression inhibited anchorage-independent growth of Caki-1 and 786-O cells (Fig. 4a,b).

### MEIS1 overexpression induces non-apoptotic death of ccRCC cells

**Fig. 5:**
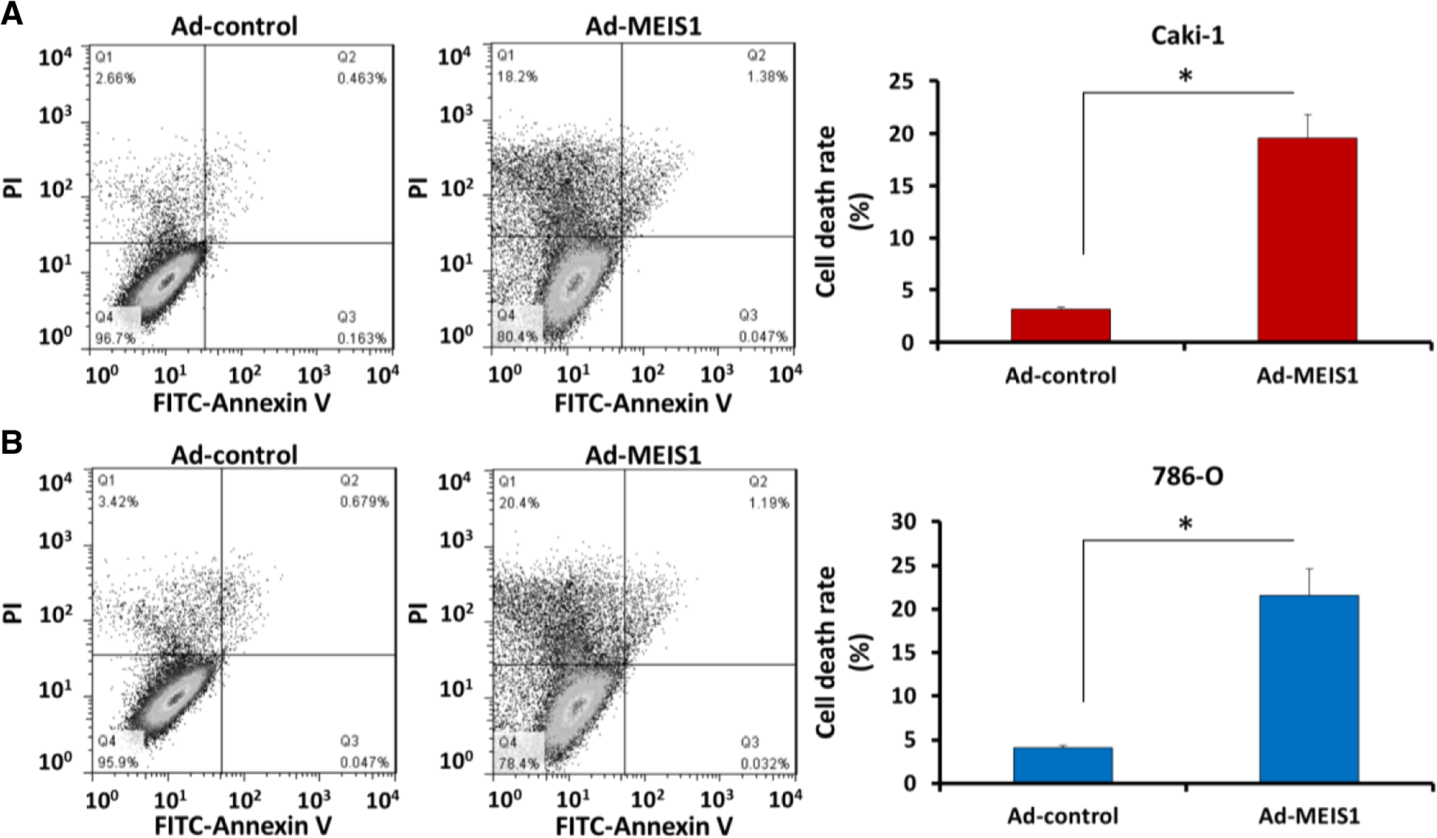
MEIS1 induces non-apoptotic cell death of ccRCC cells. **a**, **b** Representative flow-cytometer of cells stained with Annexin V and PI in (**a**) Caki-1 or (**b**) 786-O cells infected with Ad-control or Ad-MEIS1. Data were also shown as mean ± SD of three experiments with similar experiments. *p < 0.05 versus with Ad-control or Ad-MEIS1

Next, we examined whether MEIS1 participates in regulating cell death of ccRCC. As shown in Fig. 5, the proportion of non-apoptotic death increased after overexpressing MEIS1, from 3.12% to 19.58% (Caki-1), or 4.10% to 21.59 (786-O) (Fig. 5a, b).

**Fig. 6:**
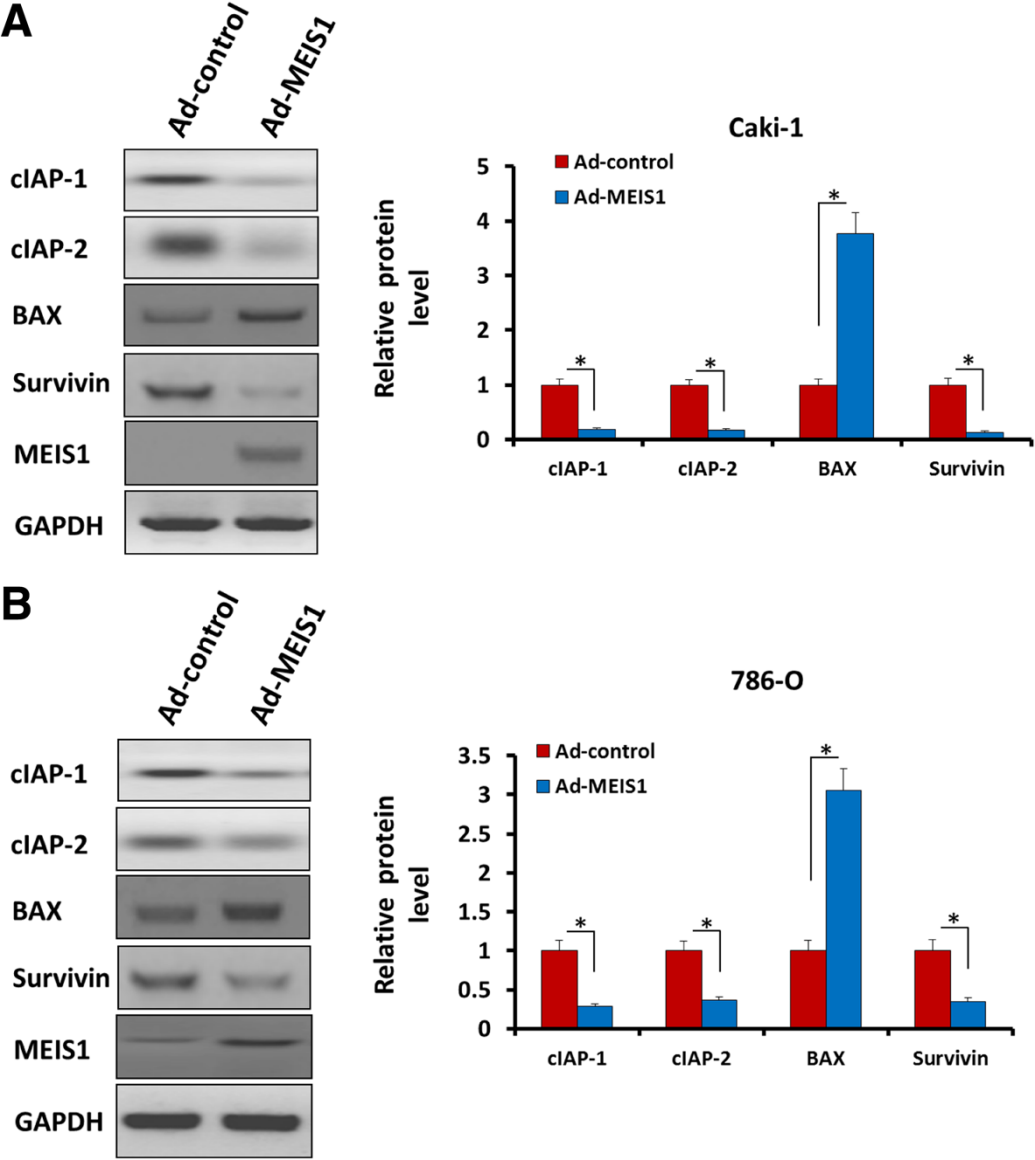
MEIS1 reduces the protein level of pro-survival and enhances the expression of pro-apoptosis regulator. Caki-1 (**a**) or 786-O (**b**) cells were infected with Ad-control or Ad-MEIS1. Then, cells were harvested for WB. Protein level of pro-survival regulators cIAP-1, cIAP-2 or Survivin and pro-apoptosis regulator BAX was detected by antibodies. GAPDH was chosen as loading controls. Relative protein level is shown as mean ± SD of three experiments with similar results. *p < 0.05 versus with Ad-control or Ad-MEIS1

To explain how MEIS1 regulates non-apoptotic cell death, we examined central components in this process. As shown in Fig. 6, overexpression of MEIS1 in Caki-1 cell reduced the expression of pro-survival proteins cIAP-1 (cellular inhibitor of apoptosis 1), cIAP-2 (cellular inhibitor of apoptosis 2) and Survivin (Fig. 6a). MEIS1 also increased the protein level of pro-apoptosis regulator BAX. Similar results were obtained from 786-O cells (Fig. 6b). These results demonstrate that the inhibitory activity of MEIS1 on ccRCC cells proliferation and survival may depend on its regulation on those pro-apoptosis related proteins.

### MEIS1 overexpression induces ccRCC cell cycle arrest at G1/S transition

**Fig. 7:**
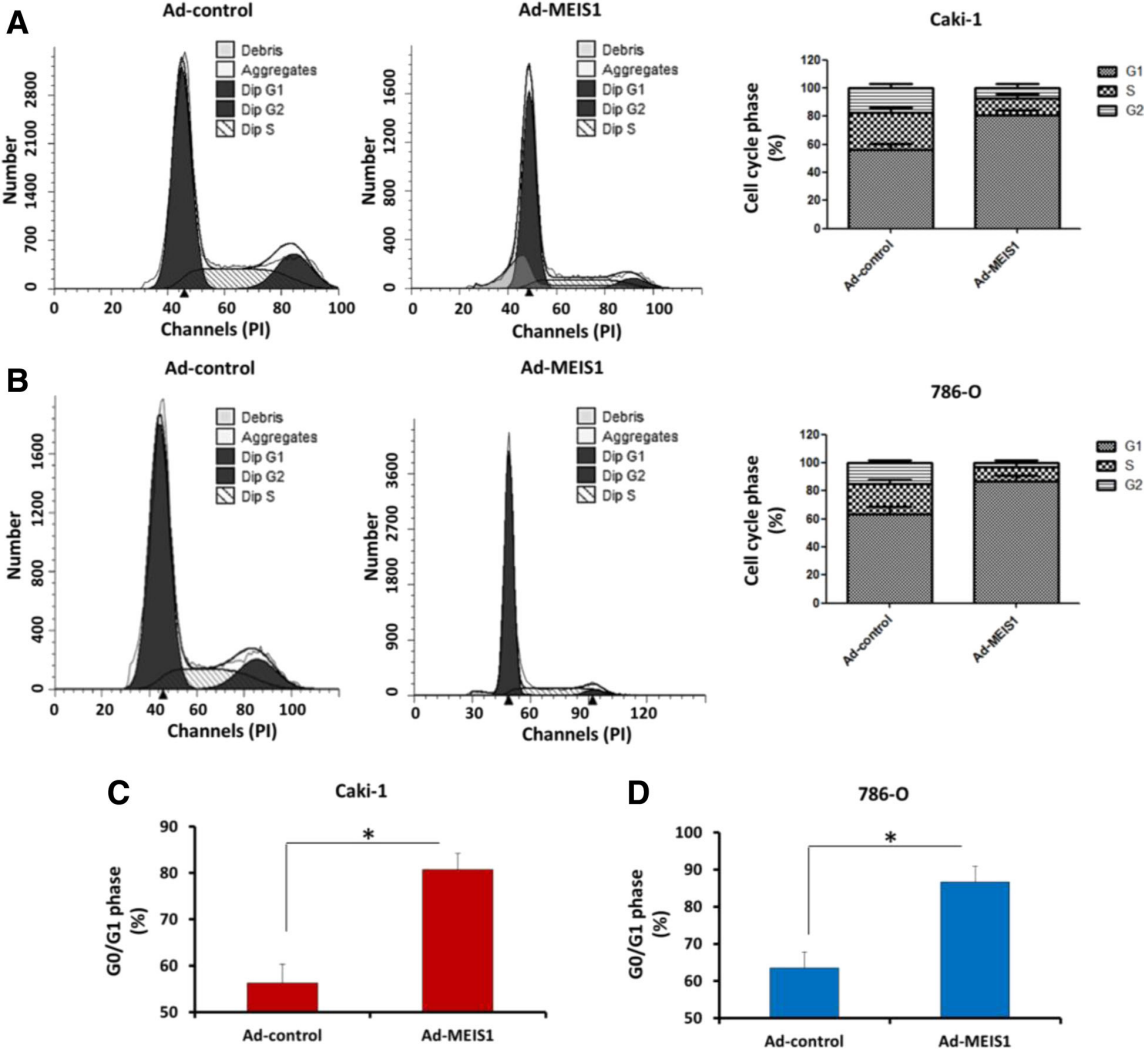
MEIS1 induces the cell cycle arrest at G1/S in ccRCC cells. **a**, **b** Cell cycle of Caki-1 (**a**) or 786-O (**b**) cells infected with Ad-control or Ad-MEIS1 were detected by flow-cytometry. The proportion of cells in each cell cycle phase was shown (**a**, **b**). The G0/G1 cells in Caki-1 (**c**) or 786-O cells (**d**) was also shown as mean ± SD of three experiments with similar results. *p < 0.05 versus with Ad-control or Ad-MEIS1

To further elucidate the mechanism by which MEIS1 negatively regulates ccRCC cells growth, we examined the effect of MEIS1 on cell cycle. Overexpression of MEIS1 in Caki-1 cells resulted in a reduction in proportion of cells in S phase (from 25.07% to 14.07%) and G2/M phase (from 14.85% to 3.25%) but an increase in proportion of cells in G0/G1 phase (from 58.25% to 75.68%) (Fig. 7a). Similar results were also obtained from 786-O cells (Fig 7b). MEIS1 overexpression increased the proportion of 786-O cells in G0/G1 phase (from 56.25% to 80.68%), and decreased S phase cells (from 26.07% to 12.07%) or G2/M cells (from 17.67% to 7.25%). These data indicated that MEIS1 could induce ccRCC cell cycle arrest at G1/S transitions.

**Fig. 8:**
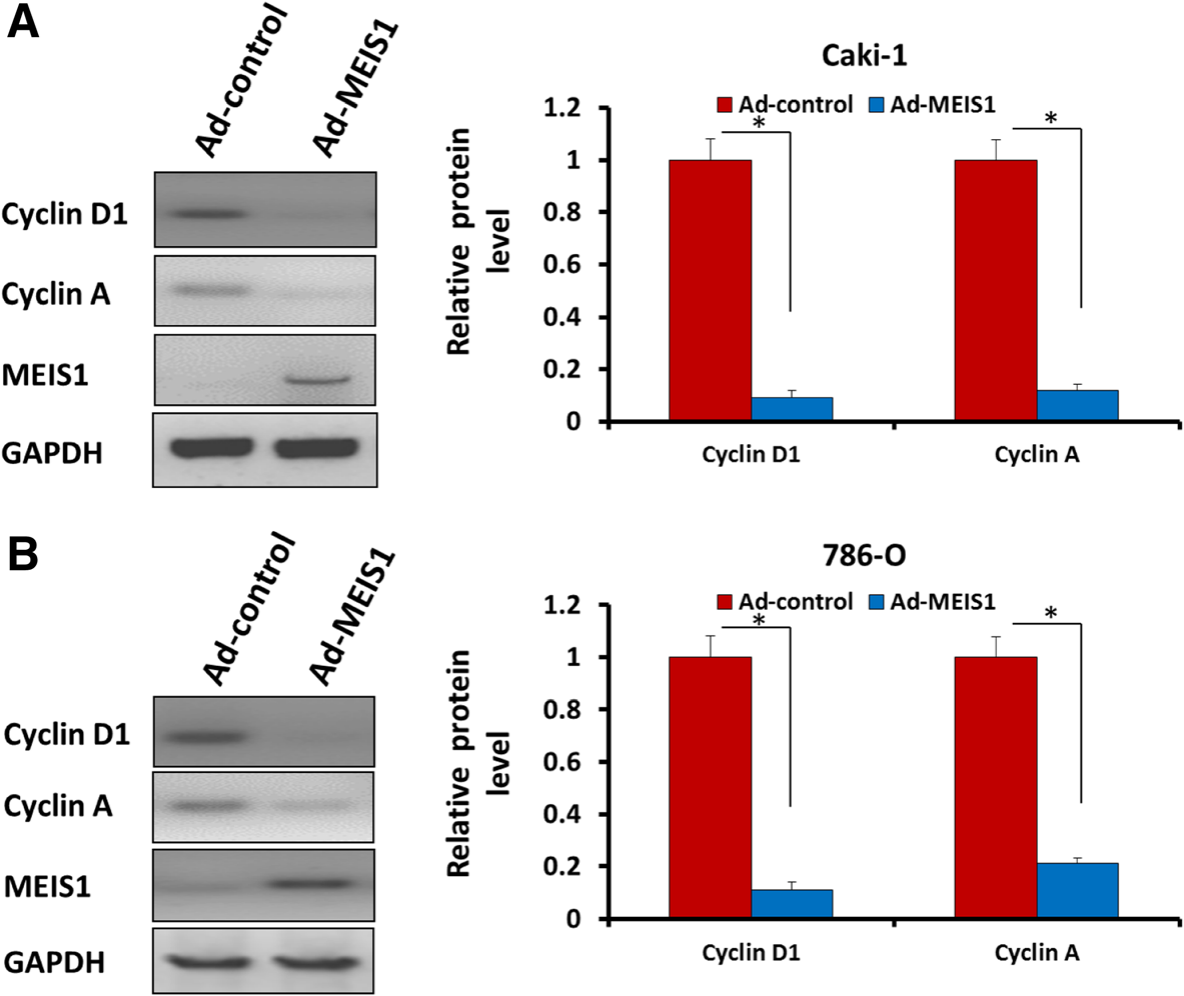
MEIS1 reduces the positive G1/S transition regulators, Cyclin D1 and Cyclin A. Representative WB for Cyclin D1 or Cyclin A in MEIS1 overexpressing Caki-1 (**a**) or 786-O (**b**) cells. Data were also shown as mean ± SD of three experiments with similar results. *p < 0.05 versus with Ad-control or Ad-MEIS1

To investigate how MEIS1 regulates cell cycle, we tested whether MEIS1 modulates the endogenous expression of positive G1/S transition regulators, Cyclin D1 and Cyclin A. As shown in Fig. 8, MEIS1 overexpression decreased Cyclin A and Cyclin D1 in ccRCC cells (Fig. 8a, b). Taken together, these data suggest that MEIS1 induces ccRCC cell cycle arrest at G1/S transitions via down-regulating cyclin A and cyclin D1.

### MEIS1 overexpression inhibits in vitro invasion and migration of ccRCC cells with decreased EMT

**Fig. 9:**
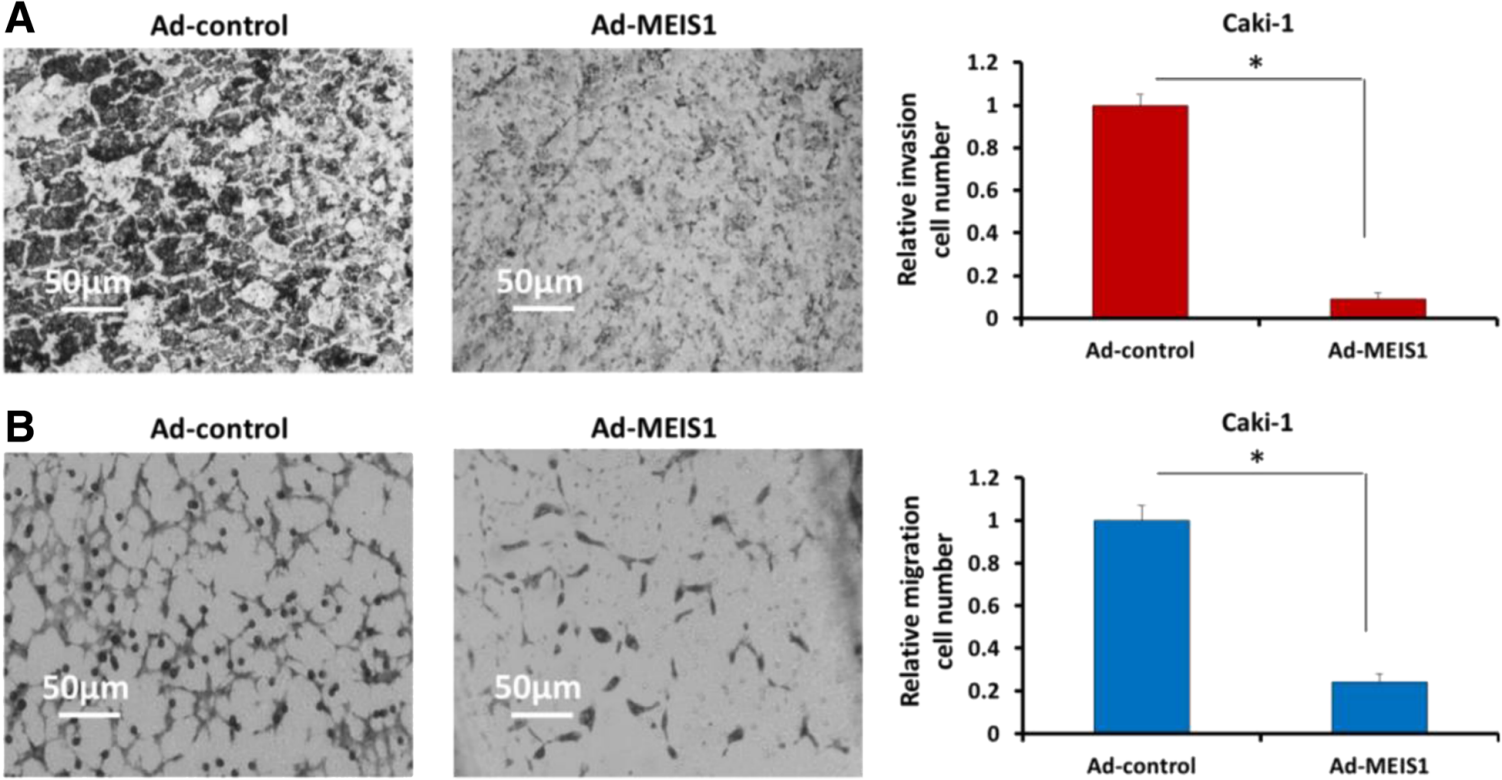
MEIS1 inhibits metastasis of high aggressive ccRCC cells Caki-1. Caki-1 cells, infected with Ad-control or Ad-MEIS1, were analyzed by transwell formation assays. Invasion (**a**) or migration (**b**) was shown as photograph or mean ± SD of triplicate measurements with similar results. *p < 0.05 versus with Ad-control or Ad-MEIS1

**Fig. 10:**
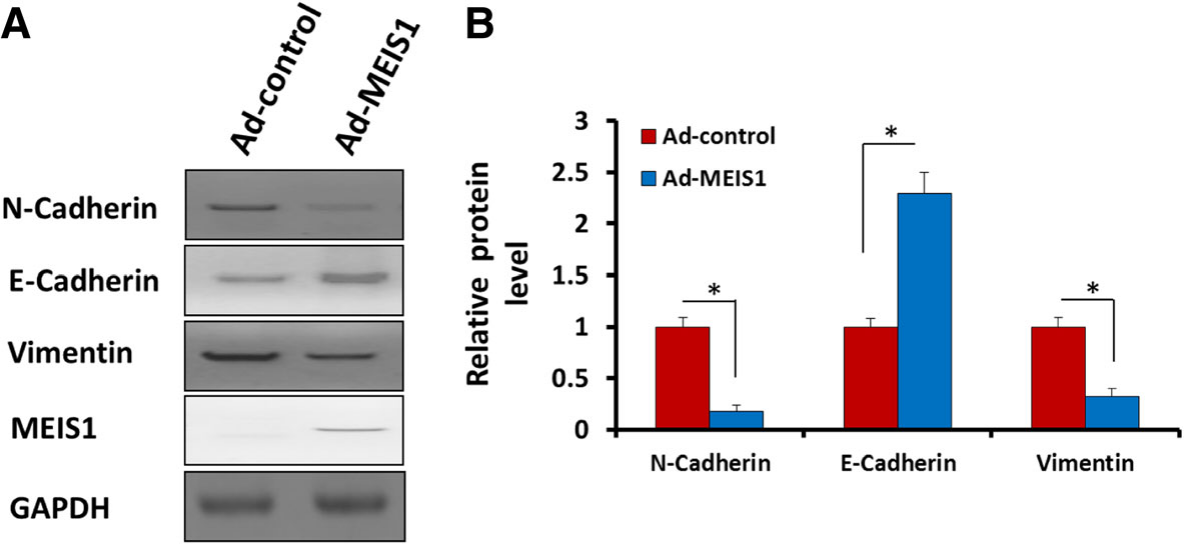
MEIS1 inhibits EMT process. Caki-1 cells were infected with Ad-control or Ad-MEIS1. The expression of epithelial marker E-cadherin and two mesenchymal markers, N-cadherin and Vimentin was detected by antibodies (**a**). Relative protein level is shown as the mean ± SD of three experiments with similar results (**b**). *p < 0.05 versus with Ad-control or Ad-MEIS1

Transwell migration assay (TMA) was used to assess the effect of MEIS1 on ccRCC cells in vitro invasion and migration. Our results indicated that overexpression of MEIS1 attenuates in vitro invasion (Fig. 9a) and migration (Fig. 9b) of the highly aggressive Caki-1 cells. Moreover, MEIS1 overexpression increased the expression of epithelial marker E-cadherin and decreased that of N-cadherin and Vimentin, two mesenchymal markers (Fig. 10a, b). These results, which were in consistent with TMA experiments, suggested that MEIS1 overexpression could inhibit the in vitro invasion and migration of Caki-1 cells via disrupting EMT process.

### MEIS1 overexpression attenuates in vivo growth of Caki-1 cells in nude mice model

**Fig. 11:**
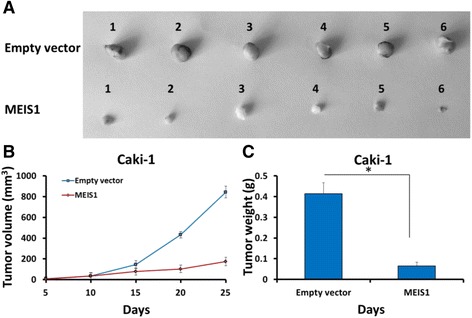
MEIS1 inhibits in vivo growth of Caki-1 cells. Caki-1 cells were infected with Ad-control or Ad-MEIS1. Next, cells were injected in to BALB/c nude mice. The effect of MEIS1 on Caki-1 in vivo growth was showed as photograph (**a**), tumor volumes (**b**) or tumor weight (**c**). *p < 0.05 versus with Ad-control or Ad-MEIS1

The effect of MEIS1 overexpression on in vivo growth of Caki-1 cells was examined in a xenograft nude mice model (Fig. 11). Infection of Ad-MEIS1 could significantly shrink the tumor volume or weight of Caki-1 compared with Ad-control. Thus, MEIS1 attenuates in vivo growth of Caki-1 cells in nude mice model.

## Discussion

For the first time, our study discovers that MEIS1 may function as a tumor suppressor in ccRCC. First, MEIS1 expression is reduced in ccRCC tissues or cell lines; second, overexpression of MEIS1 suppresses proliferation, colony formation and anchorage independent growth of ccRCC cells; third, MEIS1 overexpression induces cell cycle G1/S arrest and non-apoptotic cell death of ccRCC cells. At molecular level, MEIS1 overexpression decreases the expression of pro-survival regulators cIAP-1, cIAP-2 and Survivin; whereas increases pro-apoptosis protein BAX. Further, MEIS1 overexpression can disrupt the metastasis of Caki-1 cells and leads to decreased EMT process. These findings expand our knowledge about MEIS1 and suggest that MEIS1 may play an important role in the development and progression of ccRCC and this work may carry out its potential application in ccRCC treatment.

Cell cycle checkpoints protect genomic integrity [30]. Alterations of cell cycle related proteins, including Cyclin or CDK, would be a hallmark for multiple of human cancers [31]. The transformation of human cancers usually accompanies with the deregulation of G1/S cell cycle checkpoint controls [32]. Cyclins and their associated CDKs are the central machinery that governs cell cycle progression [21]. Of the various cyclin/CDK complexes implicated in cell cycle progression, cyclin D1/CDK4/6 and cyclin D1/CDK4/6 and cyclin A/CDK1 are of great interest, because they mediate G1/S transition [30–32]. In this study, we show that, in ccRCC, MEIS1 overexpression arrests cell cycle at G1/S transition, along with reduced expression of cyclin D1 and cyclin A. Previous reports showed that MEIS1 controls cells proliferation via regulating cyclinD1 and c-MYC level [33–35]. These results indicated that MEIS1 could be an important cell cycle regulator and attenuate cell proliferation via disrupting cell cycle progression. CDK inhibitors, e.g. P15, P16, P21, P27 or Rb may inhibit the activity of cyclin/CDK complexes and be growth inhibitor [21]. Down-regulation of these inhibitors often associates with poor prognosis of human cancers, thus it is valuable to further study whether MEIS1 regulates expression of CDK inhibitors in the future.

Recent publications provided clues that MEIS1 may participate in tumor progression, including leukemogenesis, NSCLC or nephroblastoma progression [36–38]. Among these works, MEIS1 is well-defined in modulating AML and prostate cancer progress. Also, MEIS1 would function as a tumor suppressor in some other kinds of human cancer [11–14]. It promotes the differentiation and suppresses the proliferation of non-tumor prostate epithelial cells [3, 13]. Decreased expression of MEIS1 is predicted as worse overall survival (OS) while elevated level of MEIS1 was associated with improved OS in prostate cancer [3, 13]. Our previous work also indicated that MEIS1 functions as a negative AR regulator and inhibits the ligand-dependent growth of certain kinds of prostate cancer [16]. Based on the previous discovery of MEIS1, to further reveal its function and develop potential therapy in human cancer treatment are accessible. This work discovers, for the first time, MEIS1 as a tumor suppressor in ccRCC. In this work, MEIS1 overexpression could repress in vitro invasion and migration of high aggressive ccRCC cell line Caki-1 and lead to decreased EMT, the critical step mediating cancer metastasis. Since invasion and migration are main features of malignancies, our data reveal that MEIS1 overexpression could be a potential approach to improve the cancer prognosis of advanced metastatic ccRCC.

## Conclusions

In summary, MEIS1 functions as a suppressor in ccRCC progression, supported by the fact that endogenous expression of MEIS1 reduces in ccRCC cells lines specimens Overexpression of MEIS1 significantly inhibits proliferation and apoptosis of ccRCC cells. These findings would help us to understand more about MEIS1 in cancerous cell proliferation and also provide a new potential approach to ccRCC treatment.

## Abbreviations

AML: Acute myeloid leukemia
ccRCC: Clear cell renal cell carcinoma
CDK: Cyclin dependent kinase
ECM: Extracellular matrix
EMT: Epithelial-mesenchymal transition
MEIS1: Myeloid ecotropic viral integration site 1
NSCLC: Non-small-cell lung cancer
PI: Propidium iodide
PVDF: Poly-vinylidene fluoride
TALE: Triple amino acid loop extension
